# Benefit of a single recruitment maneuver after an apnea test for the diagnosis of brain death

**DOI:** 10.1186/cc11408

**Published:** 2012-07-03

**Authors:** Marie Paries, Nicolas Boccheciampe, Mathieu Raux, Bruno Riou, Olivier Langeron, Armelle Nicolas-Robin

**Affiliations:** 1 Department of Anesthesiology and Critical Care, Groupe Hospitalier Pitié-Salpêtrière, 47-83, boulevard de l'Hôpital, 75651 Paris Cedex 13, France; 2 ER 10 UPMC, Département de Physiologie, Université Pierre et Marie Curie-Paris 6, 4 place Jussieu 75005 Paris, France; 3 Department of Emergency Medicine and Surgery, Groupe Hospitalier Pitié-Salpêtrière, 47-83, boulevard de l'Hôpital, Paris 75651 Cedex 13, France; 4 UMRS INSERM 956, Université Médecine Pierre et Marie Curie-Paris 6, 4 place Jussieu 75005 Paris, France

## Abstract

**Introduction:**

Many potential lung transplants are lost because of hypoxemia during donor management. We hypothesized that the apnea test, necessary to confirm the diagnosis of brain death in potential lung donors, was involved in the decrease in the ratio of partial pressure of arterial O_2 _to fraction of inspired O_2 _(PaO_2_/FiO_2_) and that a single recruitment maneuver performed just after the apnea test can reverse this alteration.

**Methods:**

In this case-control study, we examined the effectiveness of the recruitment maneuver with a comparison cohort of brain dead patients who did not receive the maneuver. Patients were matched one-to-one on the basis of initial PaO_2_/FiO_2 _and on the duration of mechanical ventilation before the apnea test. PaO_2_/FiO_2 _was measured before (T1), at the end (T2) and two hours after apnea test (T3).

**Results:**

Twenty-seven patients were included in each group. The apnea test was associated with a significant decrease in PaO_2_/FiO_2 _from 284 ± 98 to 224 ± 104 mmHg (*P *< 0.001). The decrease in PaO_2_/FiO_2 _between T1 and T3 was significantly lower in the recruitment maneuver group than in the control group (-4 (-68-57) vs -61 (-110--18) mmHg, *P *= 0.02). The number of potential donors with PaO_2_/FiO_2 _> 300 mmHg decreased by 58% (95% CI: 28-85%) in the control group vs 0% (95% CI: 0-34%) in the recruitment maneuver group (*P *< 0.001).

**Conclusions:**

The apnea test induced a decrease in PaO_2_/FiO_2 _in potential lung donors. A single recruitment maneuver performed immediately after the apnea test can reverse this alteration and may prevent the loss of potential lung donors.

## Introduction

Lung transplantation has become a procedure of choice for patients with irreversible, progressively disabling, end-stage pulmonary disease [1]. Among criteria for lung donors, one of the most significant appears to be arterial blood gas analysis before harvest since this parameter has been found to be significantly associated with recipient prognosis [2]. Arterial blood gas standard criteria for lung donors are a ratio of arterial oxygen tension (PaO_2_) to fractional inspired oxygen (FiO_2_) of greater than 300 mm Hg and a positive end-expiratory pressure (PEEP) of 5 cm H_2_O [3]. During the donor management and before organ procurement, many factors may damage the respiratory function of the donor. One of these factors is the apnea test, used to assess the clinical diagnosis of brain death, as a supplement to the absence of motor response to a painful stimulus and to the absence of brainstem reflexes. Usually, the patient has to be disconnected from the mechanical ventilator for 8 to 10 minutes. If there is a partial pressure of arterial carbon dioxide of 60 mm Hg or higher or an increase of more than 20 mm Hg from the baseline value, apnea is confirmed [4]. But apnea is known to be responsible for atelectasis and to decrease PaO_2_/FiO_2 _ratio in critically ill ventilated patients [5,6]. Therefore, the apnea test may possibly contribute to worsening of the respiratory function in potential lung donors.

In normal or morbidly obese patients, recruitment maneuvers (RMs) allow re-opening of atelectasis induced by apnea and paralysis during general anesthesia, through an intentional transient increase in transpulmonary pressure. RMs are associated with a significant increase in oxygenation [7,8]. Moreover, in potential lung donors, a global protective ventilator strategy that includes small tidal volumes, high PEEP, apnea tests performed without disconnection from the ventilator, and closed circuit for airway suction was recently shown to significantly increase the number of eligible and harvested lungs, although the precise role of each item has not been determined [9]. Thus, we hypothesized that the apnea test used to diagnose brain death could impair gas exchange in potential lung donors and that a single RM performed immediately after could restore it.

## Materials and methods

The study was approved by our ethics committee (Comité de Protection des Personnes se prêtant à la RechercheBiomédicale de l'HôpitalPitié-Salpetrière, Paris, France) and was performed in accordance with the Declaration of Helsinki. This study was also conducted in accordance with French law concerning multiple organ procurement. Medical management of patients and diagnosis of brain death were performed as recommended by the French conference of experts in 2005 [10] and by the American Academy of Neurology [11]. Brain death was clinically suspected with usual criteria and had to be confirmed with an apnea test, required for the diagnosis of brain death in France.

The study design was an observational, case controlled study comparing a recent historic cohort of matched control patients with prospectively included treated patients. Before June 2008, the apnea test was performed without any RM. From June 2008, a new protocol was established in our unit, providing a single RM performed immediately after the apnea test administered after the reconnection to the ventilator in potential donors. The RM was the only modification made to our lung management in brain-dead patients between the historical control period and the intervention period.

**Figure 1:**
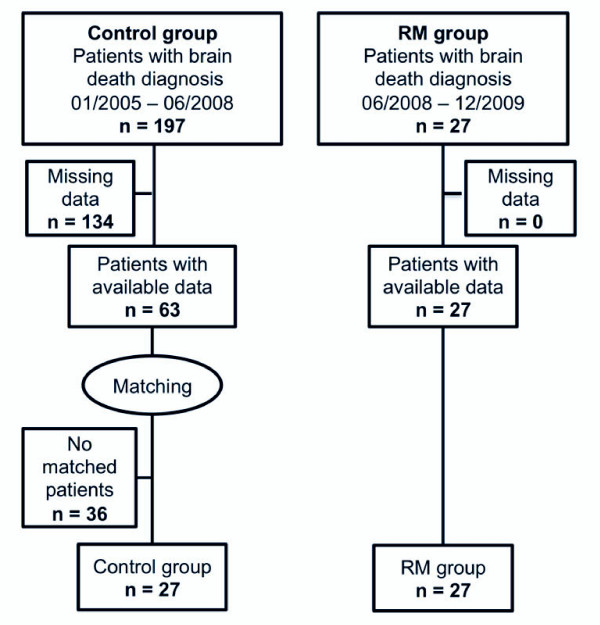
**Patient flowchart and study design**. RM, recruitment maneuver.

All patients who required an apnea test to confirm brain death between January 2005 and June 2008 were included in the historic cohort, independently of the criteria for lung donors. Patients were retained if arterial blood gas samples taken 2 hours after the apnea test were available. Blood gas samples were taken at the request of the organ procurement team and not to guide lung management of potential donors. We selected, from among these patients, a matched control group (*n *= 27, control group). After June 2008 to December 2009, all patients (*n *= 27, RM group) who required an apnea test to confirm brain death were prospectively included. Patients were excluded only if an apnea test had not been performed or achieved. In this nested case cohort study, patients in the RM group were matched one-to-one on the basis of initial PaO_2_/FiO_2 _ratio and mechanical ventilation duration (both identified as putative confounding factors) with patients who were not recruited among the cohort. Matching was performed as follows: For each case, a subset of patients was selected among a historical cohort on the basis of PaO_2_/FiO_2 _ratio and mechanical ventilation duration (± 10%). A single patient was subsequently randomly extracted from this subset of matched patients by using a random number table. A flowchart of the study is shown in Figure 1.

### Study protocol

All patients received ventilation with tidal volumes of 6 to 10 mL/kg of predicted body weight, PEEP of 5 cm H_2_O, respiratory rate adjusted to obtain normocapnia, and FiO_2 _adjusted to obtain a pulse oximetry saturation of greater than 90% (Evita XL; Dräger Medical AG and Co. KGaA, Lübeck, Germany). When required, airway suction was performed with an open circuit. Before the apnea test, the physician checked the absence of confounding variables known to interfere with brain-death diagnosis (low arterial blood pressure, hypothermia, neuromuscular relaxation, and circulating psychoactive drugs). After clinical assessment of unresponsiveness to noxious pain stimuli and abolition of brainstem reflexes, the apnea test was performed as follows: First, FiO_2 _was increased to 1 for 15 minutes for preoxygenation. At the end of the preoxygenation period, an initial arterial blood sample (T1) was taken. Then, the ventilator was disconnected for 10 minutes and a continuous flow of oxygen at a rate of 6 L/minute was administered to maintain oxygenation. The absence of chest movement indicated a diagnosis of neuronal death in the medulla oblongata. At the end of the test and just before the ventilator was reconnected with the same settings as before the test, a second arterial blood sample (T2) was taken to demonstrate a partial pressure of arterial carbon dioxide of 60 mm Hg or higher or an increase of more than 20 mm Hg from the baseline value.

In the RM group, immediately after reconnection to the ventilator, an RM was performed as follows: First, PEEP was increased from 5 to 35 cm H_2_O and the respiratory rate was decreased to 0.5 beats per minute for 40 seconds. Next, the initial ventilatory settings were re-applied. Two hours after the reconnection to the ventilator, a third arterial blood sample (T3) was taken. During the RM, we observed hemodynamic effects and oxygen saturation. Hypotension was defined by a diminution of at least 20% of the initial systolic arterial blood pressure, and desaturation was defined by a diminution of oxygen saturation of less than 90%.

### Endpoints

The main endpoint was PaO_2_/FiO_2 _ratio 2 hours after the apnea test. The secondary endpoints were PaO_2_/FiO_2 _ratio evolution during the apnea test, the proportion of potential lung donors in relation to arterial blood gas criteria, and complications of the RM in patients.

Assumptions for the sample size calculation were based on a preliminary study in 36 brain-injured patients. In these patients, we observed that PaO_2_/FiO_2 _ratio decreased from 344 ± 116 mm Hg at T1 to 276 ± 124 mm Hg at T2. To test the hypothesis that RMs prevent 80% of the decrease of PaO_2_/FiO_2 _ratio measured 2 hours after the apnea test, we calculated that 24 patients per control group or RM group would need to be studied to achieve a statistical power of 80% and an alpha risk of 5%.

### Statistical analysis

Data are expressed as mean ± standard deviation or median (25th to 75th interquartiles) for non-normally distributed variables or as number and percentage and its 95% confidence interval (CI). The normality of the distribution of the measured variables was examined with the Shapiro-Wilk test. Comparison between the two groups was performed by using the Student *t *test, the Mann-Whitney *U *test, and the Fisher exact method, as appropriate. All *P *values were two-tailed, and a *P *value of less than 0.05 was considered significant. Statistical analysis was performed by using Prism 5 software (Software MacKiev Company, Boston, MA, USA).

## Results

We prospectively included 27 patients between June 2008 and December 2009, for whom an RM was performed immediately after the apnea test (RM group). From 2005 to June 2008, we retrospectively screened 197 brain-dead patients. Among them, 63 had complete data (that is, a well-conducted apnea test and arterial blood gas taken 2 hours after the apnea test). Among these 63 control patients, 27 were definitively included by matching to the 27 RM patients (Figure 1). Baseline characteristics of the included patients in the control and RM groups are presented in Table 1.

**Table 1:** Baseline characteristics of patients in the control group and of those in the recruitment maneuver group

	Control group (*n *= 27)	RM group (*n *= 27)	*P *value
Age, years	51 ± 15	55 ± 15	0.35
Cause of brain death			
Brain trauma	4 (15%)	8 (22%)	
Stroke	18 (67%)	16 (59%)	0.38
Others	5 (19%)	3 (11%)	
Sex			
Male	19 (70%)	21 (78%)	0.76
Female	8 (30%)	6 (22%)	
Weight, kg	76 ± 14	74 ± 13	0.54
Size, cm	173 ± 10	172 ± 9	0.89
Pulmonary disease history	1 (4%)	2 (7%)	1.00
History of smoking	8 (30%)	6 (22%)	0.75
Abnormal chest x-ray	4 (15%)	7 (26%)	0.50
Mean arterial blood pressure, mm Hg	87 ± 10	90 ± 20	0.63
Administration of catecholamine	23 (85%)	24 (89%)	1.00
Ventilatory settings			
Tidal volume, mL	525 (500-550)	550 (500-600)	0.30
Respiratory frequency, per minute	12 (12-14)	12 (10-14)	0.16
Positive end-expiratory pressure, cm H_2_O	5 (5-5)	5 (5-5)	0.67
Duration of mechanical ventilation, hours	36 (22-48)	30 (23-48)	0.79
Duration of ventilation of more than 5 days	3 (11%)	4 (15%)	1.00

Data are expressed as mean ± standard deviation (SD), as median (25th to 75th interquartiles), or as number and percentage. RM, recruitment maneuver.

**Figure 2:**
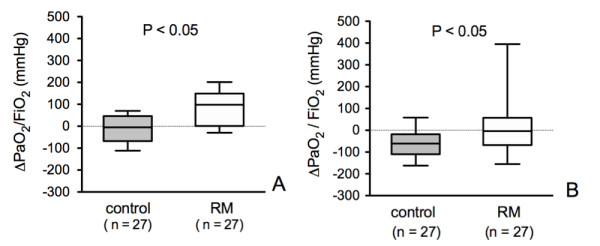
**Variation of arterial oxygen tension/fractional inspired oxygen (PaO_2_/FiO_2_) ratio**. **(a) **From the end of the apnea test before reconnection (T2) to 2 hours after reconnection (T3). **(b) **From before the apnea test (T1) to 2 hours after reconnection (T3). Values are medians with 25th and 75th percentiles (boxes) and 95th and 5th percentiles (whiskers). RM, recruitment maneuver.

During the apnea test, PaO_2_/FiO_2 _ratio decreased significantly from 291 ± 95 mm Hg at T1 to 248 ± 102 mm Hg at T2 in the control group (*P *< 0.0001) and from 277 ± 101 mm Hg at T1 to 201 ± 103 mm Hg at T2 in the RM group (*P *< 0.0001). The decrease in PaO_2_/FiO_2 _ratio between T1 and T2 was not significantly different between the two groups. After the apnea test, between T2 and T3, PaO_2_/FiO_2 _ratio did not significantly change in the control group (from 248 ± 102 to 236 ± 103 mm Hg, *P *= 0.48). In contrast, in the RM group, PaO_2_/FiO_2 _ratio increased significantly (from 201 ± 103 to 283 ± 117 mm Hg, *P *= 0.009). Consequently, the variation in PaO_2_/FiO_2 _ratio between T2 and T3 was significantly greater in the RM group than in the control group (Figure 2a). As a whole, between T1 and T3, the decrease in PaO_2_/FiO_2 _ratio was significantly lower in the RM group than in the control group (Figure 2b).

**Figure 3:**
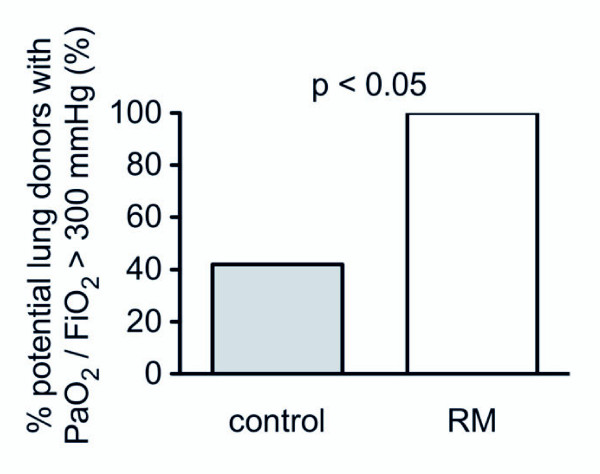
**Variation of percentage of potential lung donors according to PaO_2_/FiO_2 _criterion (between T1 and T3 from 100% at T1**. PaO_2_/FiO_2_, arterial oxygen tension/fractional inspired oxygen. RM, recruitment maneuver.

At the same time, the number of potential lung donors with a PaO_2_/FiO_2 _ratio of greater than 300 mm Hg decreased. In the whole population (*n *= 54) and before the apnea test, the number of potential lung donors (with a PaO_2_/FiO_2 _ratio of greater than 300 mm Hg) was 21 (39%, 95% CI 26% to 53%): 12 in the control group and 9 in the RM group. Two hours after the apnea test, 7 of the 12 (58%, 95% CI 28% to 85%) potential donors did not meet the criteria any more in the control group as compared with 0 of the 9 (0%, 95% CI 0% to 34%) in the RM group (*P *= 0.000) (Figure 3).

Complications of RM were studied in the 27 RM potential donors. Hypotension occurred during the RM in 15 cases (55%, 95% CI 37% to 72%), requiring infusion of 500 mL of normal saline in 7 cases. In 5 patients (19%, 95% CI 6% to 38%), this hypotension was resolving after 15 minutes and, in only one patient (3%, 95% CI 1% to 17%), was persistent after 30 minutes. One case (3%, 95% CI 1% to 17%) of desaturation was observed, and no case (0%, 95% CI 0% to 12%) of pneumothorax or bradycardia was observed.

## Discussion

Our study showed that the apnea test was associated with a marked decrease in PaO_2_/FiO_2 _ratio, which could be restored by an RM performed immediately after the reconnection to the ventilator. Importantly, in relation to arterial blood gas criteria for lung procurement eligibility defined as a PaO_2_/FiO_2 _ratio of greater than 300 mm Hg, RM prevented the loss of potential lung donors.

Around the world, there is a persistent and mounting supply-demand discrepancy for lung transplants. Selection criteria were established almost 30 years ago and continue to define the 'standard' lung donor to the present day. The traditional features are age of 55 years or less, clear chest radiography, PaO_2_/FiO_2 _ratio of 300 mm Hg or more, tobacco history of not more than 20 packs per year, absence of chest trauma, no evidence of aspiration/sepsis, absence of organisms detected by a sputum Gram stain, absence of purulent secretions at bronchoscopy, and a short period of mechanical ventilation [3,12]. Historically, the vast majority of organ donors have failed to meet these criteria, leading to lung harvest rates of only 15% to 25%, the lowest rates of all the major transplantable organs [13]. Among them, PaO_2_/FiO_2 _ratio is a criterion variably reached in brain-dead patients. In our study, on the basis of arterial blood gas criteria, only 39% of patients were eligible for lung procurement before the apnea test. In the series reported by Mascia and colleagues [9], 54% of lung donors in the control group met a PaO_2_/FiO_2 _ratio of greater than 300 mm Hg and a peak airway pressure of less than 30 cm H_2_O. This difference may be explained because Mascia and colleagues excluded patients who did not meet non-gasometric standard criteria for lung procurement, which are obviously related to PaO_2_/FiO_2 _ratio. In our study, patients were older (mean of 53 versus 45 years), the duration of ventilation before the apnea test was greater (mean of 51 versus 36 hours), and the PaO_2_/FiO_2 _ratio before the apnea test was lower (mean of 284 versus 396 mm Hg) in comparison with the study by Mascia and colleagues. Moreover, the proportion of patients who had a history of pulmonary disease or an abnormal chest x-ray was 24% in our study whereas these patients were excluded in the study by Mascia and colleagues.

During donor management and before the harvest, many factors may impair the donor's respiratory function: fluid overload, hemodynamic instability, endocrine failure, nephrogenic diabetes insipidus, inflammatory response, arrhythmias, hypothermia, coagulopathy, and infection [14]. Other factors may be atelectasis due to the use of a protective lung strategy and the apnea test, used to assess the clinical diagnosis of brain death [15].

We confirmed the deleterious effect of the apnea test on oxygenation. In our study, the apnea test was responsible for the PaO_2_/FiO_2 _ratio decrease from 265 to 225 mm Hg. Because inadequate preoxygenation was found to be associated with hypoxemia during the apnea test, we took care to increase FiO_2 _to 1 for 15 minutes before the apnea test [16]. Then, we showed that hypoxemia may occur even after adequate preoxygenation. These results are very close to those reported by Goudreau and colleagues [16] during uncomplicated apnea tests, in which the PaO_2_/FiO_2 _ratio fell from 248 to 206 mm Hg.

Consequently, two options were available to increase the supply of lungs available for transplantation: to expand the eligibility criteria or to prevent hypoxemia. Some teams have proposed decreasing the minimum tolerated value of PaO_2_/FiO_2 _ratio to 225 mm Hg or even removing this criterion in 'marginal' lungs being considered, despite controversies about the consequences [17]. Thabut and colleagues [2], reporting a series of 785 lung donors, demonstrated a higher relative risk of recipient death when donor PaO_2_/FiO_2 _ratio fell to below 350 mm Hg. On the other hand, Luckraz and colleagues [18] analyzed 362 lung transplants and found a higher 30-day mortality but not a higher overall mortality in the group of 52 donors with a PaO_2_/FiO_2 _ratio of between 225 and 300 mm Hg. More recently, Reyes and colleagues [19] found that 18% of the donors used for transplantation did not meet 'standard' gasometric criteria but that the post-transplant outcomes were similar to those of donors with a PaO_2_/FiO_2 _ratio of greater than 300 mm Hg. Nevertheless, it should be a safer strategy to improve the potential lung donors' oxygenation than to expand the criteria of eligibility for procurement.

To improve oxygenation, Noiseux and colleagues [20] proposed a lung recruitment protocol with intermittent 30-second periods of sustained inflation at 30 cm H_2_O. They reported that, with this protocol, two thirds of the lungs were suitable for transplantation after recruitment. As they did not compare the data with those of donors without a recruitment protocol, the exact benefit of the protocol remains unknown. Recently, Mascia and colleagues [9] showed that a protective ventilatory management of organ donors allowed the number of lung transplantations to be increased. The authors tested a global strategy of optimizing lung function to successfully harvest twice as many lungs according to standard eligibility criteria (including a PaO_2_/FiO_2 _ratio of greater than 300 mm Hg). The global strategy included protective ventilation (with tidal volumes of 6 to 8 mL/kg of predicted body weight), PEEP of 8 to 10 cm H_2_O, an RM performed after any disconnection from the ventilator, an apnea test performed by using continuous positive airway pressure, and a closed circuit for airway suction. Because of global protective respiratory management, it was not possible to ascribe the improvement in respiratory function to a specific modification, especially to RM. Our study was therefore designed to answer this question.

After apnea induced by general anesthesia and paralysis, RMs are effective to re-expand atelectasis and improve oxygenation in healthy subjects [7]. In morbidly obese patients, Reinius and colleagues [21] showed that a single RM followed by PEEP was efficient to reduce the amount of atelectasis and to correct the impairment of oxygenation induced by anesthesia. In brain-dead patients, we have shown that a single RM followed by PEEP might restore oxygenation impaired by an apnea test. Moreover, this strategy may improve the rate of lung procurement. Indeed, in our control group, 58% of the potential lung donors before the apnea test were lost after the apnea test. Conversely, no potential lung donor was lost in the RM group. This result is important since only 39% of our potential donors met the criteria for lung procurement, compared with 95% in the treated group of Mascia and colleagues [9], because of a different selection of potential lung donors (as detailed above).

RMs may have some potential adverse effects. In particular, excessive pressure may cause transient hemodynamic instability, especially in hypovolemic patients [22]. It is known that 80% of brain-dead patients have hemodynamic instability and that many factors may induce real or relative hypovolemia, such as diabetes insipidus, adrenal insufficiency, and sepsis [23]. Despite taking care to correct hypovolemia before an apnea test, we could not prevent all hypotensive episodes. However, these episodes were quickly resolved, either spontaneously or after fluid administration, and no serious complication such as pneumothorax, extreme bradycardia, or cardiac arrest was observed.

Our study had certain limitations that should be noted. The control group was historic. However, randomization was impossible because French law requires informed consent, which is impossible to obtain from brain-dead patients. However, the matching of patients allowed us to have comparable groups, especially under initial pulmonary conditions. In addition, our study included a relatively small number of patients, but the effect of RM, both expected and demonstrated, was so important that the power of the study remained good. Unlike investigators in a study of patients with acute lung injury [24], we did not try to detect atelectasis and effect of RM through computed tomography scans. However, 55% of our patients were hemodynamically unstable, making any move to the radiology unit potentially deleterious. Lastly, the choice of RM technique requires discussion [25,26]. We chose a sustained high-pressure inflation using a PEEP of 35 cm H_2_O for a duration of 40 seconds for the sake of simplicity. The PEEP of 35 cm H_2_O was limited by the settings possible on our ventilator. The choice of this procedure seemed to be the easiest implementation and the most reproducible by all physicians.

## Conclusions

Our study showed that the apnea test was associated with impairment in respiratory function in potential organ donors, and this may limit the eligibility for donor lung harvest. A single RM applied just after reconnection to the ventilator prevented the decrease of PaO_2_/FiO_2 _ratio and may prevent the loss of potential lung donors. Our study suggests that an RM should be routinely applied after the apnea test in potential lung donors.

## Key messages

• The apnea test, used to assess the clinical diagnosis of brain death, may be associated with impairment in oxygenation in potential organ donors and may limit the eligibility for donor lung harvest.

• A single recruitment maneuver applied just after reconnection to the ventilator prevents the decrease of PaO_2_/FiO_2 _ratio and may prevent the loss of potential lung donors.

## Abbreviations

CI: confidence interval; FiO_2_: fractional inspired oxygen; PaO_2_: arterial oxygen tension; PEEP: positive end-expiratory pressure; RM: recruitment maneuver.

## Competing interests

The authors declare that they have no competing interests.

## Authors' contributions

NB participated in study design and performed data collection. AN-R, OL, and BR participated in study design. MP performed data collection. MR made computer programs and performed primary data analysis. All authors participated in manuscript preparation and read and approved the final manuscript.
